# From hybrid to fully remote clinical trial amidst the COVID-19 pandemic: Strategies to promote recruitment, retention, and engagement in a randomized mHealth trial

**DOI:** 10.1177/20552076221129065

**Published:** 2022-09-25

**Authors:** Leigh Ann Simmons, Jennifer E Phipps, Mackenzie Whipps, Paige Smith, Kathryn A Carbajal, Courtney Overstreet, Jennifer McLaughlin, Koen De Lombaert, Devon Noonan

**Affiliations:** 1 Department of Human Ecology, 8789 University of California, Davis, Davis, CA, USA; 2 Department of Obstetrics and Gynecology, UC Davis Health, Sacramento, CA, USA; 3 Pattern Health, Durham, NC, USA; 4 Yuzu Labs, Silicon Valley, CA, USA; 5 School of Nursing, 3065 Duke University, Durham, NC, USA

**Keywords:** remote clinical trials, studies, digital clinical trials, studies, mHealth, psychology, apps, personalized medicine, pregnancy, medicine, media, obesity, lifestyle, behavior change, lifestyle, diet, lifestyle, digital health, general

## Abstract

Clinical trials worldwide were disrupted when the COVID-19 pandemic began in early 2020. Most intervention trials moved to some form of remote implementation due to restrictions on in-person research activities. Although the proportion of remote trials is growing, they remain the vast minority of studies in part due to few successful examples. Our team transitioned Goals for Reaching Optimal Wellness (*GROWell*), an NIH-funded (R01NR017659) randomized control trial (RCT; ClinicalTrials.gov identifier NCT04449432) originally designed as a hybrid intervention, into a fully remote clinical trial. *GROWell* is a digital dietary intervention for people who enter pregnancy with overweight or obesity. Primary outcomes include gestational weight gain and six-month postpartum weight retention. Strategies that we have tested, refined, and deployed include: (a) use of a HIPAA-compliant, web-based participant recruitment and engagement platform; (b) use of a HIPAA-compliant digital health platform to disseminate *GROWell* and conduct study visits (c) interconnectivity of these two platforms for seamless recruitment, consent, enrollment, intervention delivery, follow-up, and study team blinding; (d) detailed SMS messages to address initial challenges with protocol adherence; (e) email notifications alerting the study team about missed participant surveys so they can follow-up; (f) remuneration using email gift cards with recipient choice of vendor; and (g) geotargeting social media campaigns to improve participation of Black Indigenous and People of Color Communities. These strategies have resulted in screen failure rates improving by 7%, study task adherence improving by an average of 20–30% across study visits, and study completion rates of 82%. Researchers may consider some or all of these approaches in future remote mHealth trials.

## Introduction

When the COVID-19 pandemic began in early 2020, clinical trials all over the world were disrupted or delayed.^1–3^ Most behavioral health intervention trials in the U.S. have had to pivot to fully remote intervention and evaluation protocols as a result of restrictions on in-person research activities.^4–6^ Moving these trials into a fully remote format, however, was not without precedence. Prior to the COVID-19 pandemic, research had shown the promise and challenges with these pivots across all stages of research, including participant recruitment, intervention delivery, and data collection.^7^

One such recent innovation in remote trials is internet-mediated recruitment particularly using social media sites such as Facebook, Twitter, Instagram, and others. Studies have found that social media–based recruitment strategies can reach a massive audience relatively cheaply. As a result, these recruitment methods are rapidly gaining in popularity with those who conduct clinical evaluations.^8^ However, a recent review of the literature found that sampling representativeness can suffer when compared with traditional methods of recruitment.^9^ Although to remedy this, targeted recruitment for intervention trials and recruitment of “hard to reach” populations via social media can be especially effective.^9^ Other studies have found that there tends to be lower participation and adherence to study protocols in general with samples that are recruited online, especially if the intervention being evaluated is app-based^10,11^ or utilizes passively collected data.^12^ Despite these challenges, fully remote and internet-mediated recruitment strategies are likely to become even more prolific in the near future.^8^

Remote delivery of mHealth interventions also comes with its own set of strengths and challenges. For example, one of the primary strategies to increase adherence to health interventions and to study protocols is the incorporation of *personalized feedback*.^13^ In the case of fully remote and mobile app–based studies, this personalized feedback can plausibly be made automatic, eliminating delays and errors in feedback, reducing researcher burden, and increasing participant engagement with the study.^14^ However, current research is mixed on the effectiveness of this strategy. Some studies have found small positive effects on desirable data collection response behaviors,^15,16^ while others have found no effects.^17–19^

Finally, strategies for data collection in fully remote mHealth studies also have matured in the last 10 years. Active collection of participant data (e.g. collecting survey data within an mHealth app) is one such strategy. Studies again have shown mixed results for the effectiveness of these methods. Rangan et al. found that a real-time electronic dietary intake app showed good agreement with the established data collection methods (i.e. a 24-h diet recall survey).^20^ In Zapata et al.'s study of an exercise-promotion app, however, researchers found that participants engaged in the promoted exercising behaviors consistently, but did not utilize the app as a companion, and in fact saw the app as a significant *barrier* to study adherence.^21^ Passive data collection (e.g. collecting ongoing data from a Bluetooth-enabled health device) likewise has both pros and cons. Bluetooth-enabled technology, including devices such as accelerometers, GPS tools, and remote heart rate monitors, has dramatically increased as a health behavior research tool.^22^ Internet-connected food scales for measuring portion sizes and food consumed have been found to be a feasible way to collect dietary information,^23^ and adherence to daily weighing with a Bluetooth-enabled scale for those with heart failure has been shown to be quite high.^24^ However, there are significant ethical issues with passively collected health data that have yet to be satisfactorily resolved.^25^ Obtaining informed consent, data security, informational privacy, and equitable access—especially for vulnerable groups—are some of the major concerns.

Among the most challenging data collection issues that have arisen in fully remote studies has been the collection of biospecimens from participants. Some examples include the collection of blood,^26^ saliva,^27^ hair,^28^ and breastmilk.^29^ Traditionally, these data collection strategies have necessitated an in-person visit with a trained data collector, either in the participant's home or in a clinic setting.^7^ Further many of the aforementioned specimens require strict processing and storage requirements to perform required tests, such as timely centrifuging and storage on ice. It is possible for participants to appropriately self-collect biospecimens in their own home and send them to the research team,^30^ and this has proven to be indispensable for health researchers during the current pandemic.^31^ Remote training of participants and regular reminders have been shown to be critical for adherence,^30,32^ especially when reminders and training materials are culturally tailored to optimize recruitment, retention, and adherence.^33^ Video- or multimedia-based training is a particularly recent innovation that has shown great promise. Allen et al., for example, found that multimedia-based training to collect dried blood spots was widely acceptable, feasible, and produced a high percentage of useable samples for daily smokers recruited on Facebook for a fully remote study.^34^ However, unanswered questions remain about the feasibility and reliability of these methods for other populations.

Like many other researchers engaged in clinical trials,^4–6^ our team had to pivot when the COVID-19 pandemic halted in-person studies. Our trial was already utilizing a hybrid approach and we were in the pre-recruitment phase when California issued the shelter-in-place order in March 2020.^35,36^ Thus, we were able to transition to a fully remote clinical trial prior to enrolling our first participant. In this paper, we describe the process of making these transitions and lessons learned. We also discuss some of the challenges with this approach that we have remedied in part or in full.

## Methods

**Figure 1.**
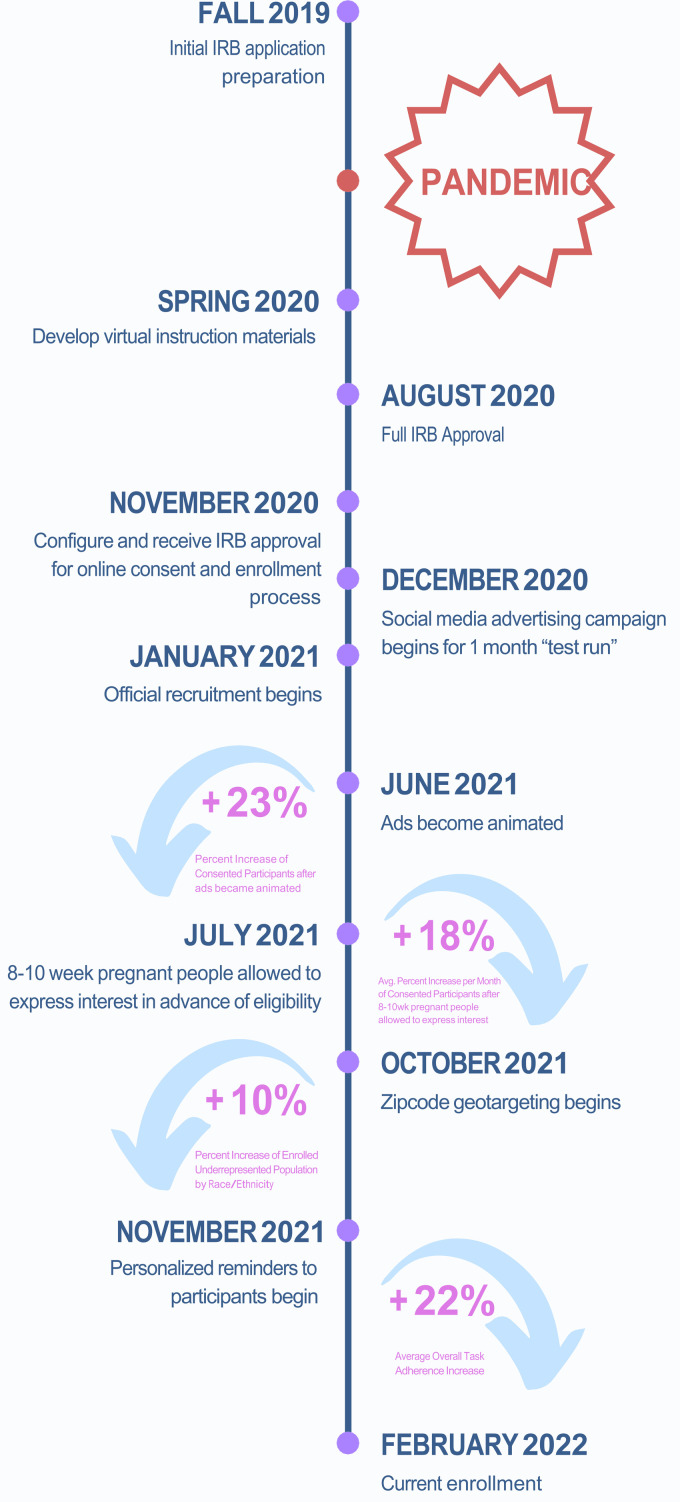
Protocol changes and associated improvements.

Goals for Reaching Optimal Wellness (*GROWell*) is an NIH-funded (R01NR017659) randomized control trial (RCT; ClinicalTrials.gov identifier NCT04449432) to test a digital dietary intervention for improving diet quality during pregnancy and the first 6 months postpartum among people who enter pregnancy with overweight or obesity.^37^ To be eligible for the study, participants must be 10–16 weeks gestation with a singleton, low-risk pregnancy, have a body mass index of 25–40 before pregnancy, living and receiving prenatal care in California, at least 12 months since a previous birth or pregnancy lasting longer than 20 weeks, non-smoking for at least 6 months, and without untreated depression or on a stable class and dose of antidepressants for at least 6 months. The study received institutional review board approval prior to study initiation and for all protocol amendments. Our primary outcomes include gestational weight gain and postpartum weight retention at 6 months post-birth. We compare the intervention to an attention control that provides pregnancy and early infancy education via weekly texts. Per our original protocol, we intended to consent in person and to conduct three in-person study visits (baseline, 36–38 weeks gestation, and six months postpartum) and two online study visits (26–28 weeks gestation and three months postpartum). With the onset of the COVID-19 restrictions on clinical research, we replaced all in-person procedures using two key technology platforms: StudyPages (Yuzu Labs Public Benefit Corporation, 2022) and Pattern Health (Durham, NC). See Figure 1 for the timeline of study activities and the effects of protocol changes. StudyPages is a HIPAA-compliant, web-based participant recruitment and engagement platform that helps academic institutions communicate about, connect with, and manage interactions with people interested in participating in research. StudyPages combine study landing pages and back-end participant management capabilities to deliver a high-touch participant interaction. Pattern Health is a digital health platform that enables clinicians and researchers to deliver and manage programs, including care plans, decisions, aids, and clinical outcomes assessments quickly and easily. StudyPages and Pattern Health use Represented State Transfer (REST) Application Programing Interfaces (APIs) to transfer data between the two systems. REST APIs enable StudyPages and PatternHealth to communicate with each other and act as a gateway between the two systems without saving client data between requests. This provides a seamless experience for the user/participant from recruitment to screening, consent, enrollment, and completion of all facets of the protocol.

### Recruitment and screening

#### Social media campaigns

Although the use of social media was always a component of our original recruitment plan, Facebook and Instagram ads became our primary means of recruitment when the pandemic halted in-person research. To this end, StudyPages developed a targeted outreach ad campaign to reach a defined audience based on demographics, location, and interests. Facebook and Instagram users who match this specific target audience may see the ad while scrolling and can click on the ad to be redirected to our landing page on StudyPages. These ads redirect prospective participants to our landing page on StudyPages (Yuzu Labs Public Benefit Corporation, 2021), the participant recruitment and engagement platform we employed for the study. To date, the average cost of the social media campaign per study participant is $105. Initially, we had static ads that used pictures representing the target population. However, to increase recruitment rates, we created animated gif advertisements to capture the attention of potential participants. We now use bright colors, sparkling stars and arrows, and movement to attempt to slow down the scrolling of social media users and have them click our study advertisement.^38^ The impact of changing the study advertisements from static to animated was on average 224 more clicks per month on the advertisements and a 23% increase in consented participants.

#### Pre-recruitment

The enrollment and baseline window for participants is 10–16 weeks gestation. With the switch to remote, we adjusted our protocol to include the use of a Bluetooth scale for weight measurements (see below in the section on data collection), which we mailed to participants with instructions on use. In the early months of this approach, we noticed that many participants would sign up in the 16–18 week window, which ended up being too late for our team to mail the scale. Additionally, we noticed many prospective participants would fail the screen because they were less than 10 weeks gestation. To not lose these participants indefinitely, we enabled a “pre-screen” process in which prospective participants who are between 8 and 10 weeks gestation can be personally followed up when they become eligible at 10 weeks. We also remind potential participants to complete their screening procedures, as needed, to fully enroll participants by 16 weeks and get the scale to them as quickly as possible. These changes increased our eligibility rate from an average of 19% of those screened being eligible to 26% of those screened being eligible, thus reducing the screen fail rate from 81% to 74%.

#### Online screening

The StudyPages platform includes a landing page that describes the study, including inclusion and exclusion criteria, participation requirements, remuneration, and contact information, as well as a link to screen for eligibility. The system is automated and enables screening and enrollment 24/7 as opposed to only when clinical research coordinators (CRCs) are on the clock. After participants take the online screening survey, they receive an automated email that redirects them to sign the consent form. Landing page visitors also are able to share the study with others or take the next step towards study participation by completing a pre-screen and signing up online. When someone signs up, the team gets notified, and the contact information is stored in the study workspace. The workspace allows study teams to track and manage interested people, communicate with, engage, and retain potential and current study participants, and streamline research workflows while maintaining compliance. Study-specific features that *GROWell* employs include SMS text messaging, Voice Over Internet Protocol (VoIP) calling, and voicemail. In general, this process allows participants to autonomously review study materials and does not restrict study engagement to time or days when the study team is available.

### Consent, enrollment, and randomization

#### Consent

In addition to recruitment and screening capabilities, the StudyPages platform provides a HIPAA-compliant online consent process, as well as consent to obtain medical records from the participants’ obstetric provider. After a person passes the pre-screen questionnaire, they have the opportunity to start the consent and medical release waiver process online. Also, an email is sent to allow the person to do this step at a later point in time. The completion of consent and waiver signature is tracked in the StudyPages workspace.

#### Enrollment and randomization

Upon completion of the consent, enrolled participants are transferred from StudyPages to Pattern Health via REST APIs. StudyPages uses the Pattern Health APIs to create a new user in the Pattern Health platform and transfers all data necessary for Pattern Health to randomize a participant. Once a user is created in Pattern Health, an email is generated inviting the user to download the Pattern Health app and continue the enrollment process.

Once the participant is appropriately linked to Pattern Health, they complete an initial demographic survey to confirm race/ethnicity and contact information, including the address to send the Bluetooth scale. Because BMI calculated using the weight from the study-provided Bluetooth scale is required for randomization, participants are randomized via the Pattern Health platform after linking their Bluetooth scale to the *GROWell* application and completing their baseline assessment.

### Data collection

#### Weight

Given that gestational weight gain and postpartum weight retention are the key study outcomes, measuring weight accurately and reliably is critical. We elected to use high-quality, calibrated Bluetooth scales, which we mail to participants. The scale connects via mobile phone to the *GROWell* app, powered by Pattern Health. Although participants are directed to weigh themselves at each study visit, they may step on the scale when they choose (and many have), and *GROWell* captures these weights if their mobile phone is nearby. Although not initially part of our study design, regular weighing may impact results,^39^ and we will assess this in our final data analysis.

#### Pregnancy and birth records

Given that we are recruiting throughout California, at the time of consent we have participants sign a records request for us to obtain their prenatal and delivery medical records. These data will be used to calibrate the weights we obtain using the Bluetooth scale, as well as to evaluate potential clinical mediators or moderators of outcomes.

### Blinding

Our original protocol was designed as a double-blind study with different clinical research coordinators conducting initial and follow-up visits to retain blinding. With the shift to a fully remote trial, PatternHealth added support to the backend application for study personnel to remain blinded to randomization status. The PatternHealth console allows study teams to manage participation across users. In unblinded studies, the administrative team can see the participant's study arm, enrollment details, and their adherence to study tasks in addition to all study data collected. In order to support the *GROWell* study, PatternHealth implemented the ability to hide the study arm and all study data outside of the participants’ demographic details so that *GROWell* study team members remain blinded. However, it is important to keep the study team members informed of participants’ study progress for payment and follow-up purposes. To support the team, StudyPages implemented email notifications that alert the team when a participant is due for study payment for completing a milestone, or when a milestone is missed and a participant requires follow-up. These notifications enable the study team to manage participants without unblinding their data.

### Adherence and retention

Given the lack of human contact in this study, we have been consistently monitoring adherence and retention and making minor adjustments to address issues that arise. To date, we have made two key changes. First, we noticed that some participants were confused about the specifics of what they were supposed to do at the start of the study, and this resulted in missed baseline surveys. To remediate this, we initiated a detailed SMS message introduction through the StudyPages workspace and a handout, both of which outline what study participation looks like. This has resulted in the majority (99%) of newly enrolled participants completing the demographics and baseline survey at the correct time.

As with any longitudinal study, completion of study visits can wane over time, and the additional lack of human connection may increase the likelihood of missed study visits and/or loss to follow-up. To improve rates of adherence and reduce attrition rates, we instituted an email reminder to the study team for each participant when the study visit is due, and when the study visit is missed. This has increased overall study task adherence by 22 percentage points to greater than 80% to date.

Consistent with most clinical research studies, *GROWell* employs participant remuneration that increases over time for the completion of online study visits. In the original protocol, participants were going to receive a check; however, this delays receipt and requires signed paperwork. Instead, we pay participants using an online gift card service, Tango Card, Inc., that allows participants to choose the vendor for which they would like to redeem their gift card. The service has more than 100 online vendors to choose from, and payments are released immediately when processed by the study team.

### Inclusion of Black, Indigenous, and people of color communities (BIPOC)

Our recruitment goal for this study includes 50% White participants and 50% BIPOC. At approximately one-third of our sample size goal, we were recruiting approximately 65% White participants and 35% BIPOC participants. To address this gap, we initiated two key remedies. First, we are trialing the use of geotargeting with our social media ads. Specifically, we are increasing ads in zip codes that have a majority of residents who identify as BIPOC. Second, we have deployed *GROWell* in Spanish, given that in California, Spanish is the second most common language spoken after English, with approximately 25% of the state's population preferring Spanish as their primary language. We continue to track the effects of these adjustments on recruitment rates, and the most recent data show that we are now recruiting approximately 51% White participants, 44% BIPOC participants, and 5% who did not report.

## Discussion

In the wake of the COVID-19 pandemic, we employed a number of unique strategies to conduct *GROWell* as a fully remotely trial. First, from recruitment through study completion, *GROWell* remains a primarily automated system, thereby increasing efficiency and access for potential and existing participants. Being able to have participants take an online screening survey, then receive an automated email that redirects them to sign the consent form is exponentially faster than scheduling an in-person visit and reviewing the consent form during that visit or over the phone and also relatively novel compared to many digital intervention studies that still require some human contact, even if the recruitment and/or consent are online or electronic.^40,41^ Although still a relatively rare approach, we are not the first to have a fully automated and online recruitment and consent; this method has been shown to be a powerful way to access hard-to-reach populations.^42^ Second the system can be working on screening and enrollment 24/7 compared to only when clinical research coordinators (CRCs) are on the clock. CRCs can spend the time they would otherwise be recruiting and enrolling to focus instead on more time-intensive study tasks, like personalized follow-up. Third, text-message follow-up is quicker than only having the option to call or email each participant and increases reach in times when phone calls or email responses may not be feasible (e.g. participant is working or breastfeeding a baby). Fourth, a text-based welcome to the study that provides clear instructions about how to get started and outlines expectations for the study overall seems to increase participants’ engagement and willingness to complete future tasks assigned. Fifth, study staff can work from any location, thereby minimizing turnover and maintaining continuity. Sixth, participants can reside far from our medical center, thereby decentralizing this trial and expanding its reach to increase equitable access to studies for those who live far away or reside in more rural locations. Seventh, our remote trial helps to address time and travel barriers to participation by eliminating the need for participants to travel far distances to participate in the multiple study visits, which is especially difficult for those with transportation limitations and who visit the medical center infrequently. Relatedly, the reduced need to travel is a cost savings for the research team. Our hybrid approach would have required CRCs to travel to multiple university-affiliated clinics across a four-county region that spans approximately 145 square miles. Lastly, we anticipate that as we hone our online recruitment efforts and fully expand to Spanish-speaking participants, we will enroll a more diverse sample. This ultimately may lead to better overall representation in the study and more useful data that may deepen our understanding of the use of *GROWell* for improved dietary quality, pregnancy weight gain, and postpartum weight loss.

We also have identified some challenges that we are either in the process of remedying and/or will consider their roles in the post-implementation analysis. First, it is unclear whether participants are more or less likely to complete the online study visits when they are all remote/online. We are competing for attention during smartphone use with social media, games, and other user experiences that seek to hold their attention. That said, to date baseline and follow-up surveys are completed by participants 87% of the time on average, which is equivalent or better than anticipated for a fully remote trial.^4,43–45^ Second, it is unclear the degree to which using the Bluetooth scale for weight measurements will impact our outcomes, because regular weighing may improve weight loss or weight loss maintenance.^39^ We will need to consider this in our post hoc analyses. Third, some participants may view a fully remote trial as less personable and would prefer to interact face to face with study personnel.^46,47^ However, other individuals may prefer this approach, and a qualitative exploration of those with better and/or worse adherence or those who remained versus dropped out will provide additional information. Fourth, only the most tech-savvy individuals could be enrolling in our trial, which means that we may be exacerbating the digital divide.^48^ That said, the vast majority (upwards of 90%) of people of childbearing age have and regularly use mobile phones and other internet-capable devices, and the familiarity with technology is only increasing with every generation.^49,50^ Therefore, this concern could be overstated and be less of an issue within 5–10 years. Fifth, while our application can be used either from cell phone tower or internet connections, technologies that require one or the other limit participation of certain subpopulations. Sixth, while mHealth is expanding exponentially because of the pandemic, data security is always a concern, and our team had to manage several layers of extra security given the large volume of the private health information we were receiving. Participants use a token and date of birth authentication method to log in to the application. In addition, users can set up a PIN, fingerprint, or face id before opening the app. All notifications to study team members communicating adherence/compliance updates provide context and a link to the users (e.g. a participant in the *GROWell* study has completed their 26–28-week visit), but no participant data are included to ensure that no data are transmitted via email. The issue of data security could skew our participant pool, given that some prospective participants may be wary of using digital technology for a study about their health and health behaviors. Lastly, concerns regarding ethical issues that arise from passive data collection, including ownership of data, secondary use of data that has been collected for a specific purpose, informational privacy, and equity of access should be considered.^25^ Adherence to an ethical framework to ensure patient safety and protect patients’ rights is, therefore, an important feature of mHealth interventions and the clinical trials that evaluate them.

## Conclusion

The lessons learned from the pivot of *GROWell* to a completely remote clinical trial in the wake of COVID-19 provide valuable information to the research community about implementing future remote clinical trials. Remote clinical trials have real potential to not only increase representation and reduce participant travel and study visit burden but also bring with them challenges in implementation and participant retention. Research teams should consider remote recruitment, intervention deployment strategies, personalized feedback, and remote data collection procedures at the study planning phase and tailor methods to best engage and meet the needs of the population of focus. When considering hybrid recruitment approaches to traditional in-person or completely remote trial procedures, personnel and cost factors also need to be considered. For example, in some cases, conducting a hybrid trial may be cost-prohibitive when considering the infrastructure needed for remote methodology and additional personnel for in-person work. Remote clinical trials, especially for mHealth studies, have the potential to expand access and improve health outcomes. The pros and cons of this approach should be weighed for all future trials to maximize the benefit to study participants and to ensure a broader representation of groups historically underrepresented in clinical research.
